# Synthesis and optical properties of WS_2_ nanotubes with relatively small diameters

**DOI:** 10.1038/s41598-023-44072-z

**Published:** 2023-10-08

**Authors:** Md. Ashiqur Rahman, Yohei Yomogida, Abdul Ahad, Kan Ueji, Mai Nagano, Akane Ihara, Hiroyuki Nishidome, Mikito Omoto, Shigeki Saito, Yasumitsu Miyata, Yanlin Gao, Susumu Okada, Kazuhiro Yanagi

**Affiliations:** 1 Department of Physics, Tokyo Metropolitan University, Hachioji, Tokyo 192-0397 Japan; 2 Department of Physics, Comilla University, Cumilla, 3506 Bangladesh; 3 Department of Physics, Graduate School of Science and Technology, University of Tsukuba, Tsukuba, Ibaraki 305-8571 Japan

**Keywords:** Electronic properties and materials, Organic-inorganic nanostructures, Two-dimensional materials

## Abstract

Tungsten disulfide (WS_2_) nanotubes exhibit various unique properties depending on their structures, such as their diameter and wall number. The development of techniques to prepare WS_2_ nanotubes with the desired structure is crucial for understanding their basic properties. Notably, the synthesis and characterization of multi-walled WS_2_ nanotubes with small diameters are challenging. This study reports the synthesis and characterization of small-diameter WS_2_ nanotubes with an average inner diameter of 6 nm. The optical absorption and photoluminescence (PL) spectra of the as-prepared nanotubes indicate that a decrease in the nanotube diameter induces a red-shift in the PL, suggesting that the band gap narrowed due to a curvature effect, as suggested by theoretical calculations.

## Introduction

Tungsten disulfide (WS_2_) nanotubes^1^ are cylindrical nanotubes composed of WS_2_ sheets, which are semiconducting transition metal dichalcogenides (TMDCs), and have attracted significant attention over the past few decades owing to their unique electronic, optical, and optoelectronic properties^2,3^. Unlike carbon nanotubes, WS_2_ nanotubes exhibit solely semiconducting properties regardless of how they are rolled^4^ and show advantages for applications such as sensors^5^, field effect transistors^6–8^, optoelectronic devices^9,10^, and thermoelectric devices^11^. Owing to their chiral nanotube structures, WS_2_ nanotubes exhibit several interesting properties, such as nonreciprocal conduction^8,12^ and bulk photovoltaic properties^12, 13^. It has been theoretically predicted that the electronic structure of WS_2_ nanotubes depends on the nanotube structure, which is characterized by the nanotube diameter^14–17^ and wall number^18^. In particular, WS_2_ nanotubes with small diameters are expected to exhibit significant band structure modulation due to curvature effects^15,19,20^, leading to unique properties different from those of WS_2_ sheets and WS_2_ nanotubes with large diameters. Thus, an important goal in this field is to clarify the correlation between the properties and nanotube structures experimentally. Most of the studies to date have been performed on relatively large-diameter multiwall nanotubes (~ 100 nm)^21–23^ and have revealed structure-dependent properties, such as size-dependent exciton-polaritons^17^, structure sorting optical properties^16^, and light-matter interactions^14^. In contrast, very few studies^24^ have investigated the properties of small-diameter WS_2_ nanotubes because of their challenging synthesis (owing to the high folding energy of WS_2_ sheets). Although the synthesis of MoS_2_ nanotubes with small diameters outside carbon nanotubes and inside boron nitride nanotubes has been reported^25,26^, the structure–property relationship in small-diameter WS_2_ nanotubes has not been experimentally investigated to date.

One approach to synthesize TMDC nanotubes is the chalcogenization of transition metal oxide nanowires, which has been used for large-scale synthesis^22^. In this process, the oxide nanowires are synthesized and then converted into nanotubes by sulfurization. Therefore, to synthesize small-diameter nanotubes with few walls, it is necessary to synthesize small-diameter oxide nanowires. Recently, several studies have attempted to reduce their diameter and narrow their diameter distribution by modifying the synthesis of oxide nanowires; as a result, nanotubes with diameters of 70 nm^27^ and 20 nm^28,29^ have been fabricated from oxide nanowires synthesized from chemical vapor-deposition (CVD) and solvothermal methods, respectively. In addition, although the structure distribution is not known, nanotubes with diameters of 8–25 nm have been obtained by this approach^24^. Therefore, these approaches exhibit high potential for the production of small-diameter TMDC nanotubes.

This study reports the synthesis of small-diameter WS_2_ nanotubes by the chalcogenization of structure-controlled tungsten oxide nanowires fabricated via CVD. Detailed structure characterization confirmed that the as-prepared multi-walled WS_2_ nanotubes showed the innermost diameter of 6 ± 3 nm, and the wall number of 5 ± 2. The utilization of single-crystalline small-diameter tungsten oxide nanowires as the starting material enabled the synthesis of high-crystallinity small-diameter WS_2_ nanotubes. The optical absorption and photoluminescence (PL) characteristics of the samples were used to elucidate the effects of the nanotube diameter on the bandgap. The PL of the as-prepared small-diameter WS_2_ nanotubes was red-shifted from the PL of WS_2_ flakes and large-diameter WS_2_ nanotubes, indicating a bandgap reduction, in agreement with the results of theoretical calculations^15,19^.

## Results and discussion

In this study, WS_2_ nanotubes were synthesized on Si/ SiO_2_ (300 nm) or quartz substrates under Ar gas at a sulfurization temperature of 600 °C from tungsten oxide nanowires. The nanowires were first grown on silicon substrates by CVD^30,31^ and then transferred onto Si/ SiO_2_ or quartz substrates for sulfurization^28,29^. Details of the synthesis are provided in Methods section. As mentioned previously, the synthesis of small-diameter tungsten oxide nanowires is a prerequisite for the synthesis of small-diameter WS_2_ nanotubes. In this study, as described in the Methods section, we used WO_2.9_ powder for tungsten source and the synthesis of the oxide nanowires was performed in Ar + H_2_ (4%) atmosphere.

Typical low and high-magnification transmission electron microscopy (TEM) images (Supplementary Fig. S1(a,b) in the Supplementary information (S.I)) and the diameter distribution shown in Supplementary Fig. S1(c) in S.I. indicate that the tungsten oxide nanowires used as precursors for nanotube synthesis are single crystalline and exhibit an average diameter (with standard deviation) of 13 ± 5 nm. The average diameter of these tungsten oxide nanowires is slightly smaller than that of previously reported solvothermally synthesized nanowires (~ 17 nm)^28^. Field-emission scanning electron microscopy (FESEM) images of the nanowires (Supplementary Fig. S2(a) in S.I.) indicate high-density nanowire growth on the substrate, while X-ray diffraction (XRD) patterns (Supplementary Fig. S2(b) in S.I.) indicate that the nanowires comprise the monoclinic W_18_O_49_ phase, consistent with literatures^30–35^.

**Figure 1:**
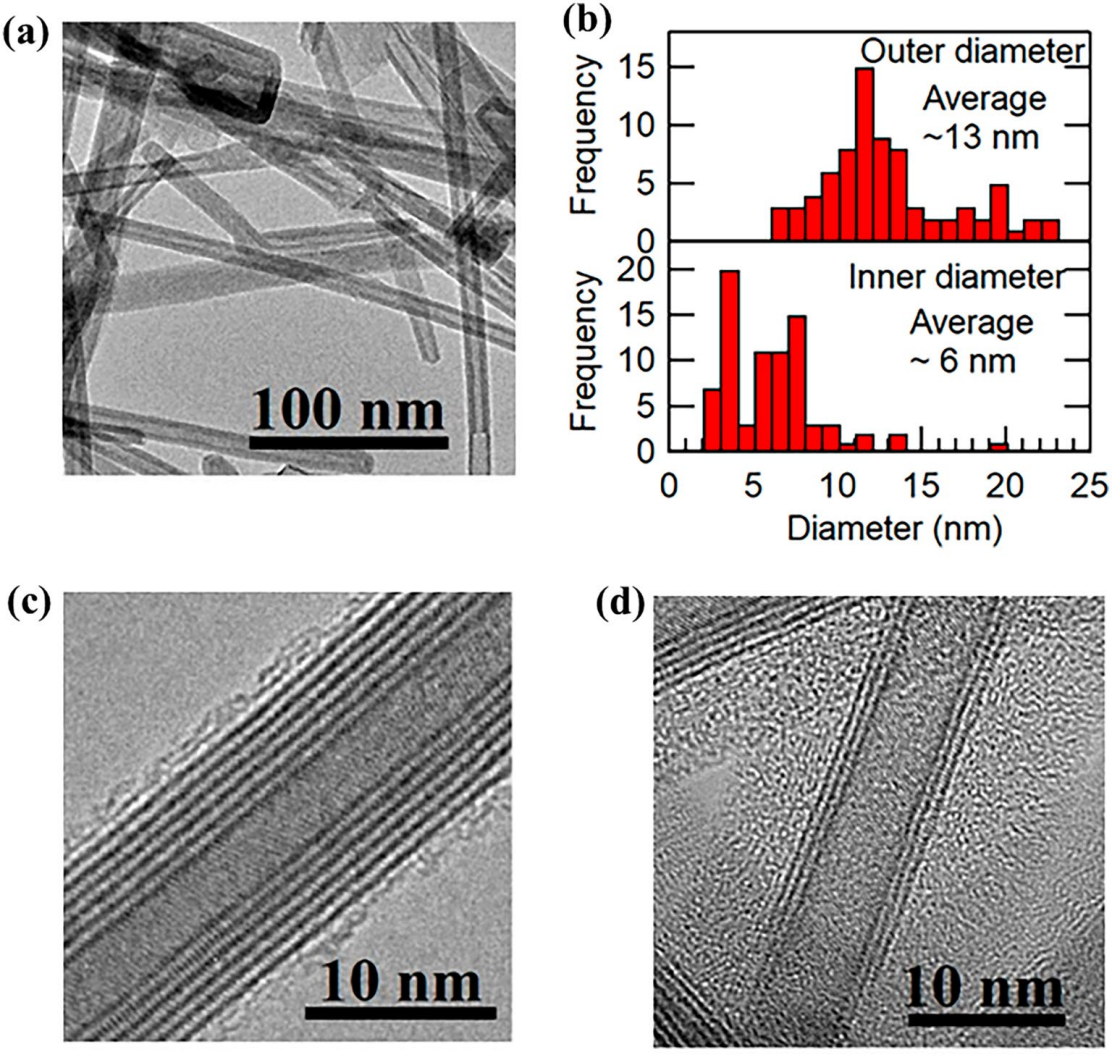
TEM observation of the as-synthesized WS_2_ nanotubes. (**a**) Low-magnification TEM image of the WS_2_ nanotubes. (**b**) Histogram of the outermost and innermost tube-diameter distributions of the WS_2_ nanotubes. (**c**), (**d**) High-magnification TEM images of the WS_2_ nanotubes.

TEM images of the as-synthesized WS_2_ nanotubes indicate hollow, multi-walled structures (Fig. 1a). The outermost and innermost diameters of the nanotubes were statistically analyzed to evaluate the nanotube structure distribution (Fig. 1b). The average outermost and innermost diameters (with standard deviations) of the as-prepared nanotubes are 13 ± 4 nm and 6 ± 3 nm, respectively, and the average wall number (with standard deviation) is 5 ± 2 (Supplementary Fig. S3 in S.I.). Figure 1c,d show high-magnification TEM images of the WS_2_ nanotubes. The average diameter of the as-prepared WS_2_ nanotubes is significantly smaller than that of commercially available WS_2_ nanotubes (average diameter ~ 100 nm, purchased from Nanomaterials Co., Supplementary Fig. S4 in S.I.)^21–23^ and slightly smaller than that of WS_2_ nanotubes derived from solvothermally synthesized tungsten oxide nanowires (average diameter: ~ 20 nm)^28,29^. The diameters of the as-prepared WS_2_ nanotubes are compared with those of previously reported WS_2_ nanotubes^28,29^ in Supplementary Table S1. Raman spectroscopy and energy-dispersive X-ray spectroscopy (EDS) (Supplementary Fig. S5 in S.I.) were used to confirm the elemental composition and quality of the WS_2_ nanotubes. X-ray photoelectron spectroscopy (XPS) (Supplementary Fig. S6 in S.I.) indicates that the WS_2_ nanotubes comprise the 2H–WS_2_ phase^36^, while FESEM images (Supplementary Fig. S7(a) in S.I.) indicate that the presence of impurities like nano-flakes is negligible. Additionally, XRD analysis (Supplementary Fig. S7(b) in S.I.) confirms that the WS_2_ nanotubes comprise the 2H–WS_2_ phase. Moreover, the absence of XRD signals corresponding to tungsten oxide nanowires in the spectrum of the as-prepared WS_2_ nanotubes indicates almost complete conversion of oxide nanowires to WS_2_ nanotubes during chalcogenization. Fast Fourier transform (FFT) patterns of the TEM images were used to investigate the chirality of the WS_2_ nanotubes (with three walls) (Supplementary Fig. S8 in S.I.). FFT patterns indicate three different chiral angles; therefore, the different walls of the nanotube exhibit different chirality.

Here, the influence of the nanotube structure on the optical properties of the as-prepared nanotubes was investigated. The absorption spectra of WS_2_ flakes (with a thickness of 20–40 nm), commercially available WS_2_ nanotubes with an average diameter of 100 nm (L-WS_2_ NTs; purchased from Nanomaterials Co.), and WS_2_ nanotubes with an innermost diameter of 6 ± 3 nm (S-WS_2_ NTs; synthesized in this study) were measured and analyzed. For optical measurements, the S-WS_2_ NTs and L-WS_2_ NTs were directly dispersed in methanol, while the WS_2_ flakes were dispersed in methanol after liquid-phase exfoliation (see S.I.)^37,38^.

**Figure 2:**
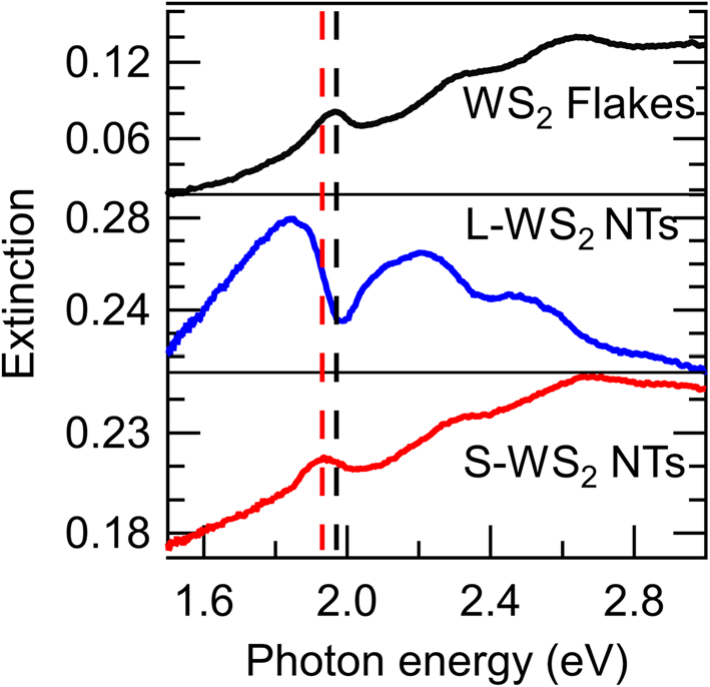
Extinction spectra of WS_2_ flakes, large-diameter (L-WS_2_ NTs) and small-diameter (S-WS_2_ NTs) nanotubes. The black (upper panel), blue (middle panel), and red (lower panel) curves show the extinction (absorption) spectra of WS_2_ flakes, L-WS_2_ NTs, and S-WS_2_ NTs, respectively.

The optical extinction (absorption) spectra of the WS_2_ flakes, L-WS_2_ NTs, and S-WS_2_ NTs, recorded by a conventional UV–VIS spectrometer (UV-3600, Shimadzu), are shown in Fig. 2. The A, B, and C excitons of the WS_2_ flakes are observed at 1.97 eV, 2.33 eV, and 2.65 eV, respectively, in good agreement with the literature^39,40^. The spectrum of the L-WS_2_ NTs, unlike that of the WS_2_ flakes, exhibits dips close to the position of the A and B excitons, owing to the polaritonic nature of the nanotubes (due to the tube diameter of the L-WS_2_ NTs)^14^. This phenomenon is caused by strong exciton–photon coupling in the optical-cavity structure of nanotubes with diameters of ~ 100 nm. We examined the optical absorption spectra of L-WS_2_ NTs by using an integrating sphere in the UV‒VIS spectrometer and confirmed that the A exciton peak of L-WS_2_ NTs is located at 1.97 eV (Supplementary Fig. S9 in S.I.), which is similar to that of WS_2_ flakes. Theoretical calculations indicate that this polaritonic effect disappears when the nanotube diameter decreases below 40 nm, owing to difficulties in nanotube-cavity optical confinement^14^. Therefore, polariton-like dip structures are absent in the absorption spectrum of S-WS_2_ NTs, which exhibit outermost and innermost diameters of 13 nm and 6 nm, respectively. A similar phenomenon is observed in sorted small-diameter WS_2_ nanotubes^16^. Due to the suppression of the polaritonic effect in small-diameter nanotubes, the absorption peak at 1.92 eV in S-WS_2_ NTs can be attributed to the A exciton, showing a slight redshift compared to the A exciton peak in the WS_2_ flakes.

**Figure 3:**
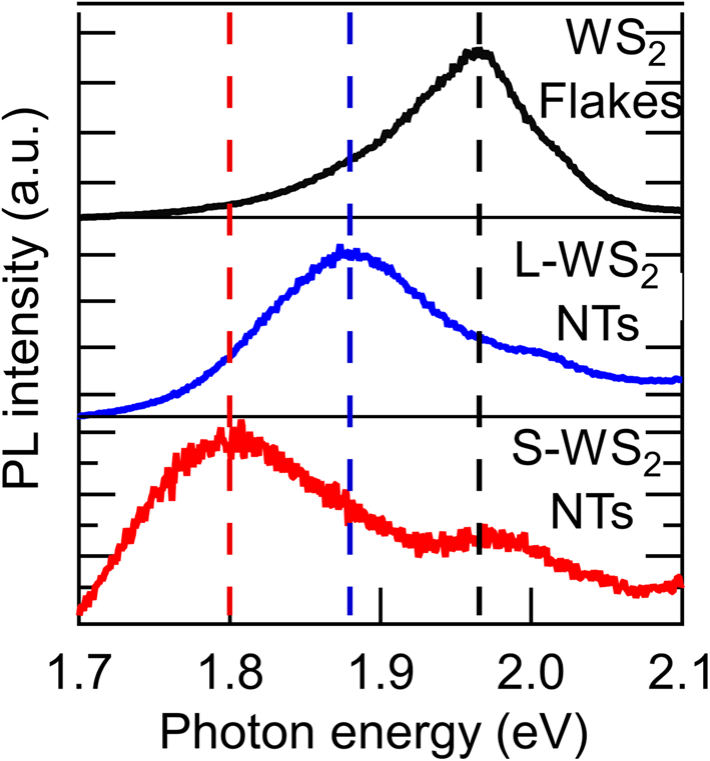
Photoluminescence (PL) spectra of WS_2_ flakes, large-diameter commercial nanotubes (L-WS_2_ NTs), and the as-synthesized small-diameter nanotubes (S-WS_2_ NTs). The black (upper panel), blue (middle panel), and red (lower panel) curves show the PL spectra of WS_2_ flakes, L-WS_2_ NTs, and S-WS_2_ NTs, respectively.

To further investigate the effects of the WS_2_ nanotube structure on their optical properties, PL spectra were investigated. We placed the samples on quartz substrates by drop-casting the dispersions in methanol. The PL measurements for all samples were carried out with a 532-nm excitation laser. Figure 3 shows the PL spectra of the WS_2_ flakes, L-WS_2_ NTs, and S-WS_2_ NTs; all the background signals are removed to highlight the PL signals from the samples. All the samples exhibit PL peaks; however, the PL intensities of the multi-walled WS_2_ nanotubes and flakes are weak owing to indirect bandgap structures. The PL peak of the WS_2_ flakes is observed at 1.97 eV, which is consistent with the A-peak position of its absorption but is blue-shifted to the reported PL peak energy, 1.91 eV, of the mechanically exfoliated WS_2_ bulk sample^41^. This blue-shift behavior is commonly reported in liquid phase exfoliated WS_2_^42^. The PL peak of L-WS_2_ NTs (1.88 eV) is slightly red-shifted from that of the WS_2_ flakes. Remarkably, the PL peak position of the S-WS_2_ nanotubes is located at the smallest photon energy (1.80 eV).

To understand this trend of PL peaks, the bandgap energies of WS_2_ monolayers and WS_2_ nanotubes with different diameters were theoretically calculated. The geometric and electronic structures of planar and tubular WS_2_ were investigated by density functional theory with generalized gradient approximation^43,44^. The calculated band gap energies are summarized in Table 1. The bandgap energies of the monolayer, (28,28) nanotube (diameter: 5.4 nm), and (17,17) nanotube (diameter: 3.4 nm) are 1.963, 1.962, and 1.900 eV, respectively. Details of the calculated band structures are provided in Supplementary Fig. S10 in S.I. Theoretical calculations indicate that the bandgap decreases as the nanotube diameter decreases, in agreement with previous publications^15,19,20,45^. It is known that this band-structure narrowing is caused by strain and curvature effects^15,19,20,45^.

**Table 1:** Calculated bandgap energies of the WS_2_ monolayer and nanotubes.

Structure	Bang gap [eV]
	Theoretical calculation
Monolayer	1.963
(28,28) nanotube (Diameter 5.4 nm)	1.962
(17,17) nanotube (Diameter 3.4 nm)	1.900

Calculations indicate that bandgap narrowing is negligible when the nanotube diameter is greater than 5 nm. Therefore, for L-WS_2_ NTs, whose diameter is approximately 100 nm, we neglect the influence of the narrowing of the band gap on the redshift of PL compared with the PL of the WS_2_ flakes. We assume that the slight red-shift of the PL of L-WS_2_ NTs could be attributed to the lower polariton state formed by exciton-light coupling by the nanotube cavity, as similar PL has been reported in strong coupling single walled carbon nanotube microcavities^46^. In contrast, the polariton effect, which did not influence the optical absorption of S-WS_2_ NTs, was negligible in the small-diameter S-WS_2_ NTs. Theoretical calculations (of this study and in the literature^15,19,20,45^) predict bandgap narrowing for nanotubes with diameters of ~ 3 nm. The as-synthesized S-WS_2_ NTs, with an innermost diameter of 6 ± 3 nm, contain 3 nm diameter nanotubes on the inner-wall sides. In contrast to single-walled carbon nanotubes^47^, as the diameter of TMDC-NTs decreases, the bandgap reduces. This indicates that PL from the smallest (innermost-wall) nanotubes in the multi-walled WS_2_ nanotubes contributed to the observed redshifted PL spectrum of the S-WS_2_ NTs.

As the optical absorption characteristics of WS_2_ nanotubes with a diameter of ~ 10 nm predominantly influence the absorption spectrum of S-WS_2_ NTs (with an outermost diameter of 13 ± 4 nm), the optical absorption of S-WS_2_ NTs is similar to that of WS_2_ flakes. Contrarily, as PL occurs due to energy transfer and relaxation processes from the lowest excited state in nanotubes, the smallest-diameter nanotube (i.e., the innermost-wall tube) contributes predominantly towards light emission. Therefore, the PL of S-WS_2_ NTs is red-shifted from the PL of WS_2_ flakes.

The red-shift of PL is sometimes observed by formation of trions in WS_2_ monolayers^48^. However, this phenomenon does not explain the large PL red-shift (> 100 meV) observed in this study. Therefore, the observed redshift would be majorly caused by the bandgap narrowing effect. In inorganic fullerenes like WS_2_ with small layer numbers (< 5), the quantum-size effect in the direction perpendicular to the S–W–S layer induces a blue-shift in the optical absorption of the A exciton^49^. However, the PL spectra of WS_2_ flakes with a series of different layer numbers^41,50^ indicate that the PL-peak position remains almost unchanged on decreasing the number of layers from 10 to 3. The nanotubes synthesized in this study contained an average of five layers; therefore, such quantum effect was assumed to negligibly influence the PL red-shift of the as-synthesized samples.

In this study, small-diameter WS_2_ nanotubes were synthesized by the sulfurization of tungsten oxide nanowires through the CVD method. The as-prepared WS_2_ nanotubes showed an innermost diameter of 6 ± 3 nm and a mean wall number of 5. The relationships between the optical properties and nanotube diameter were investigated and a clear red-shift of the PL in the small-diameter WS_2_ nanotubes was observed. The red-shift of the PL was attributed to bandgap narrowing, in agreement with the results of theoretical calculations. The synthesis of small-diameter TMDC nanotubes reported in this study could guide future studies to clarify the basic properties of WS_2_ nanotubes, facilitating their optoelectronic applications.

## Methods

### Synthesis of WS_2_ nanotubes

To synthesize WS_2_ nanotubes, first, tungsten oxide nanowires were synthesized by temperature-controlled CVD under vacuum, according to previous studies^30,31^. Among tungsten oxide compositions, reduced tungsten oxide W_18_O_49_ is known to exhibit a tendency of uniaxial growth and contribute to the synthesis of small-diameter nanowires. Thus, in this study, to synthesize oxide nanowires in a more reducing atmosphere than that in the previous studies^30,31^, the tungsten oxide source was changed from WO_3_ to WO_2.9_, and the synthesis atmosphere was changed from air to Ar + H_2_ (4%). The nanowire synthesis was carried out using a three-zone furnace. A quartz boat with WO_2.9_ powder (3 g, 99.99%, Alfa Aesar) was placed in a quartz tube and positioned upstream/midstream of the furnace, and a quartz boat with Si substrates was positioned downstream of the furnace. During synthesis, the system pressure was maintained at 0.6–0.9 Pa with a vacuum pump while supplying 0.08 sccm of Ar/H_2_ (Ar:H_2_ = 96:4). The temperatures of the upstream/midstream and downstream zones were set at 880 °C and 580 °C, respectively. The temperatures were maintained for 6 h and were cooled naturally to room temperature. For the growth of the tungsten oxide nanowires, several substrates can be put into a furnace for CVD, and the amount of our synthesized tungsten oxide nanowires was approximately 15 ± 3 µg on a substrate (area: 1 cm × 2 cm).

The as-synthesized tungsten oxide nanowires were converted into WS_2_ nanotubes via typical conversion mechanisms reported in the literature^51–53^. S atoms were trapped at the W_18_O_49_-nanowire oxygen vacancies, and the nanowires were continuously converted to amorphous WS_3_ with a transition to the 2H–WS_2_ phase^53^. Details of the experimental setup are provided below. The sulfurization of the obtained nanowire samples was carried out using two tube furnaces, as in previous studies^28,29^. To prevent undesirable side reactions of Si substrates during sulfurization, the as-prepared nanowire samples were dispersed in ethanol and transferred onto Si/ SiO_2_ or quartz substrates. A ceramic boat with the nanowire samples on the substrates, preferentially annealed at 400 °C under high vacuum, was placed in a quartz tube and positioned downstream of the furnaces, and a ceramic boat with sulfur powder (99.99%, NewMet) was positioned upstream of the furnace. Under Ar flow (200 sccm), the furnace temperature for the nanowire samples was raised to a set temperature of 500–650 °C. After the furnace temperature reached the set temperature, the furnace temperature for S powder was raised to 250 °C, and S vapor was supplied. The furnace temperature was maintained for 1 h and then rapidly cooled to room temperature.

### Structural characterization

Structures and elemental compositions of the tungsten oxide nanowires and the as-prepared small-diameter WS_2_ nanotubes were evaluated by TEM with EDS (JEM-3200FS, JEM2100F, and JEM2010F, JEOL Ltd.), FESEM (JSM-7100F JEOL Ltd.), XRD (Rigaku SmartLab) with Cu K_α1_ (1.5406 Å) radiation, XPS (JPS-9010, JEOL Ltd.) and Raman spectroscopy (532 nm excitation, WItec).

### Theoretical calculation

All calculations were conducted using the STATE program package based on density functional theory^54,55^. The exchange–correlation potential of the interacting electrons was treated by the generalized gradient approximation with the Perdew–Burke–Ernzerhof functional^56^. An ultrasoft pseudopotential was used to describe the interaction between the valence electrons and ions^57^. The valence wave functions and deficit charge density were expanded in terms of plane-wave basis sets with cutoff energies of 25 and 225 Ry, respectively. Brillouin-zone integration was performed with 9 × 9 and 9 × 1 k-meshes for planar and tubular WS_2_, respectively. Atomic coordinates of planar and tubular WS_2_ were optimized until the force was less than 1.33 × 10^−3^ Hartree/bohr.

### Supplementary Information

Supplementary Information.

## Data Availability

The datasets generated during and/or analyzed during the current study are available from the corresponding author on reasonable request.
