# Supplementary material for: Synthesis and optical properties of WS2 nanotubes with relatively small diameters
Source: Sci Rep. 2023 Oct 8;13:16959. doi: 10.1038/s41598-023-44072-z (PMC10560667; doi:10.1038/s41598-023-44072-z)
Supplement: Supplementary file 1 — Supplementary Information. [file 41598_2023_44072_MOESM1_ESM.pdf]

Supplementary Information for

Synthesis and optical properties of WS_2 nanotubes with relatively small diameters

Md. Ashiqur Rahman[1,2], Yohei Yomogida[1], Abdul Ahad[1,2], Kan Ueji[1], Mai Nagano[1], Akane Ihara[1], Hiroyuki Nishidome[1], Mikito Omoto[1], Shigeki Saito[1], Yasumitsu Miyata[1], Yanlin Gao[3], Susumu Okada[3], and Kazuhiro Yanagi[1*]

[1]*Department of Physics, Tokyo Metropolitan University, Hachioji, Tokyo 192-0397, Japan*

[2]*Department of Physics, Comilla University, Cumilla-3506, Bangladesh*

[3]*Department of Physics, Graduate School of Science and Technology, University of Tsukuba, Tsukuba, Ibaraki 305-8571, Japan*

E-mail: yanagi-kazuhiro@tmu.ac.jp, yomogida@tmu.ac.jp

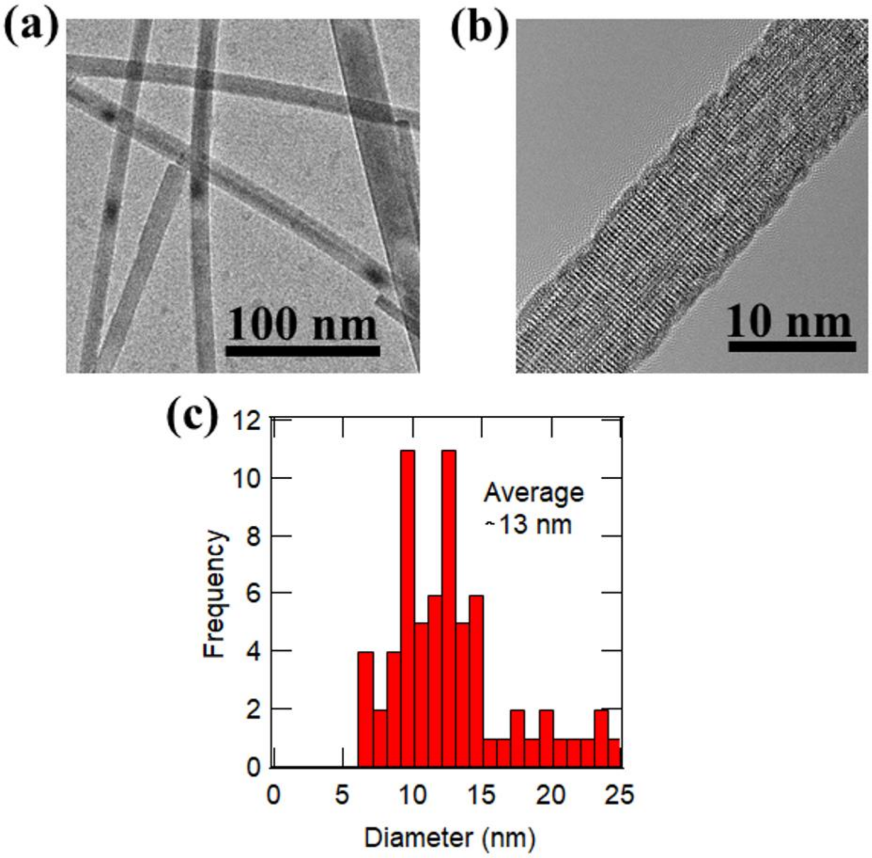

Fig. S1. Evaluations of the tungsten oxide nanowires obtained by CVD method. (a) Low-magnification TEM image of tungsten oxide nanowires. (b) High-magnification TEM image of tungsten oxide nanowires. (c) Histogram of diameter distribution of CVD nanowires.

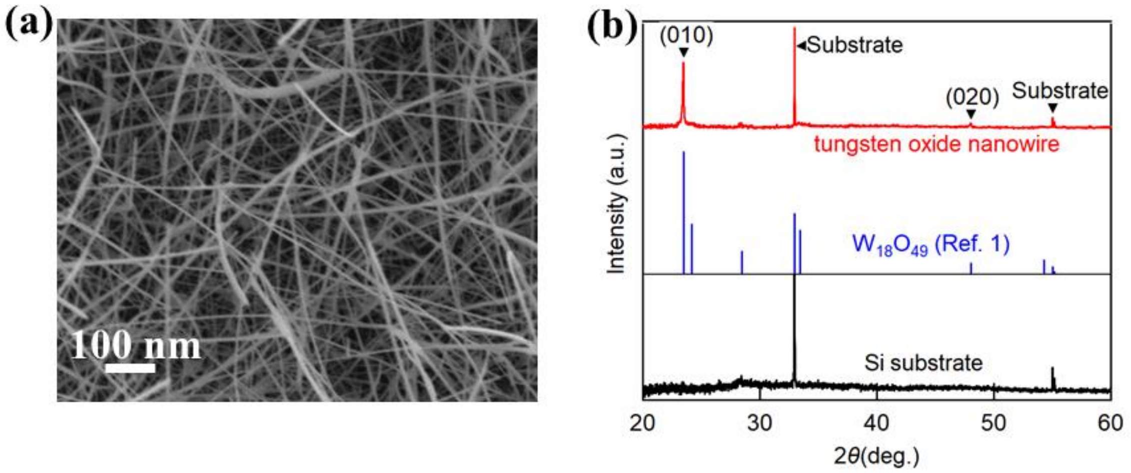

Fig. S2. (a) FESEM image of the tungsten oxide nanowires on the Si substrate. The tungsten oxide nanowires were grown in relatively high-density on the substrate. (b) The XRD patterns of the tungsten oxide nanowires on the Si substrate, $W_{18}O_{49}$, and the Si substrate are shown. The pattens for the nanowire and the substrate were obtained using Rigaku Smart Lab ($CuK_{\alpha 1}$, 1.5406 Å), and the XRD patten for $W_{18}O_{49}$ is depicted using the data in a literature [1]. The XRD pattern of the nanowires exhibits two characteristic peaks at 23.45 and 47.35° position representing (010) and (020) plane reflections of the monoclinic $W_{18}O_{49}$ phase [1]. The strong peak (010) diffraction indicates a preferred growth of $W_{18}O_{49}$ nanowires along the [010] direction, which is consistent with the previous literatures [2-7].

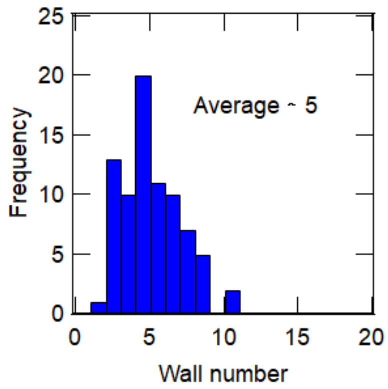

Fig. S3. Wall number histogram of synthesized WS_2 nanotubes.

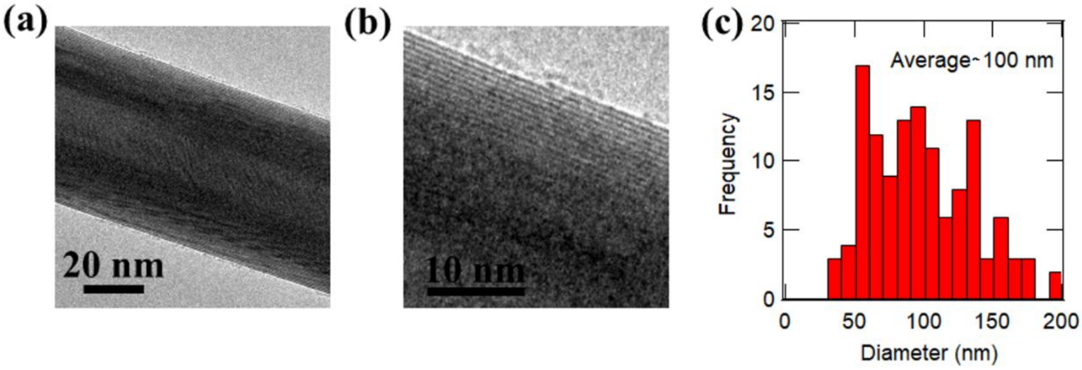

Fig. S4. (a,b) Typical TEM image of large diameter (commercial: L-WS_2 NT) WS_2 nanotubes. (c) The diameter distribution of the large diameter WS_2 nanotube.

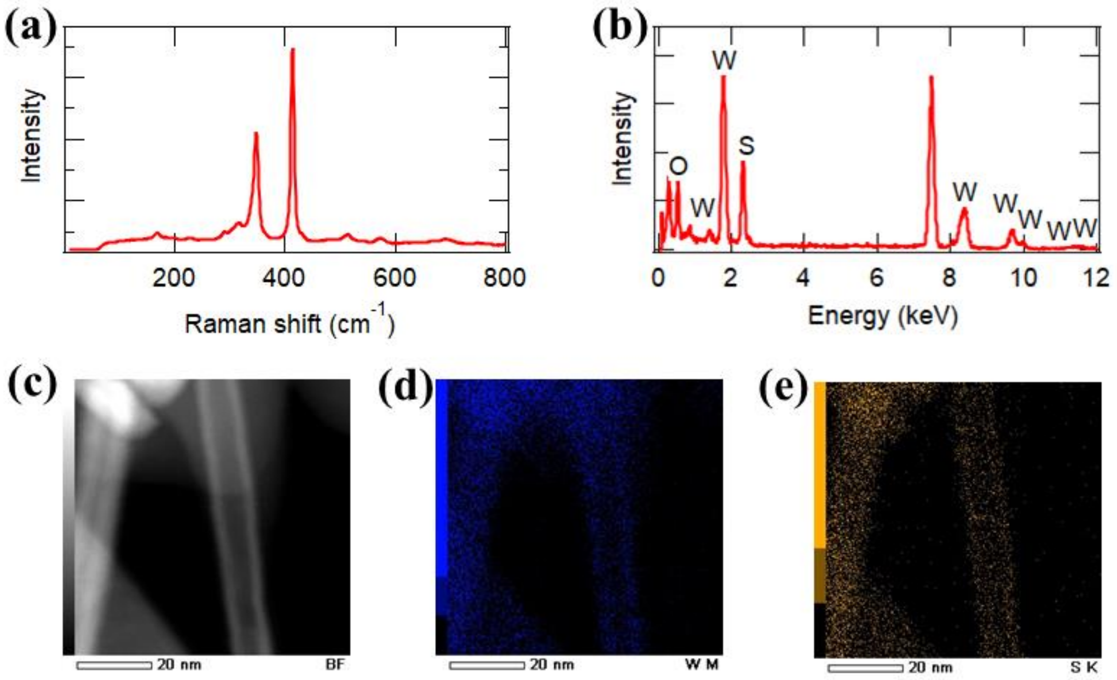

Fig. S5. (a) Raman and (b) EDS spectra of the synthesized WS_2 nanotubes. (a) The Raman peaks are assigned to the peaks from WS_2 (350 cm^{-1} for E_{2g}, 420 cm^{-1} for A_{1g}, 580 cm^{-1} for A_{1g}+LA, and 700 cm^{-1} for 4LA). (b) The EDS spectrum shows that the W/S ratio is about 1/2. The STEM-EDS mapping data of the WS_2 nanotubes are also shown; (c) STEM image, the spatially resolved W (d), and S (e) mapping.

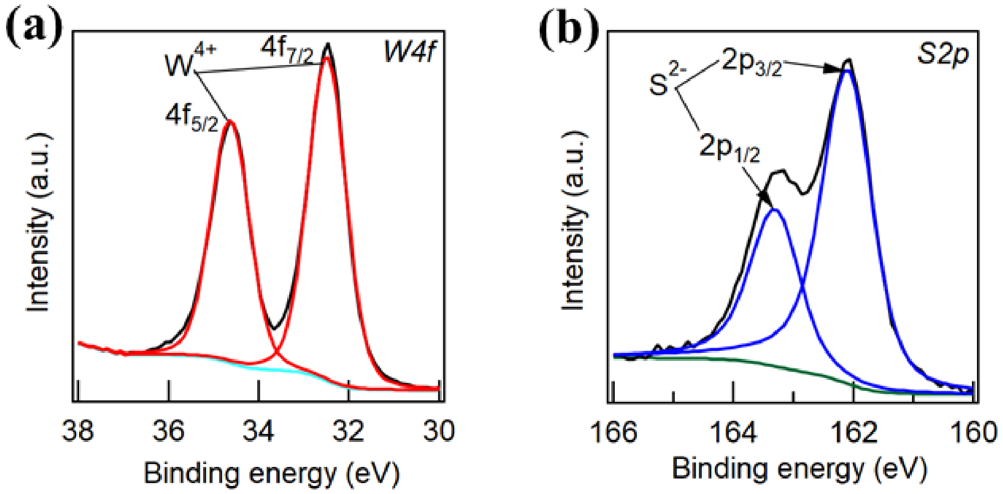

Fig. S6. The XPS data of the WS_2 nanotubes. (a) The deconvolution of the W4f core-level spectrum with peaks corresponding to W^{4+} state, and (b) S2p with S^{2-} state of WS_2 nanotubes. The data was taken by JPS-9010 (JEOL Co.) system. The $W4f_{7/2}$ and $W4f_{5/2}$ peaks at 32.48 and 34.63 eV correspond to W^{4+} state. The $S2p_{3/2}$ and $S2p_{1/2}$ peaks at 162.00 and 163.10 eV correspond to S^{2-} state. The XPS spectra of the WS_2 nanotubes indicate that the nanotubes are in the 2H-WS_2 phase [8].

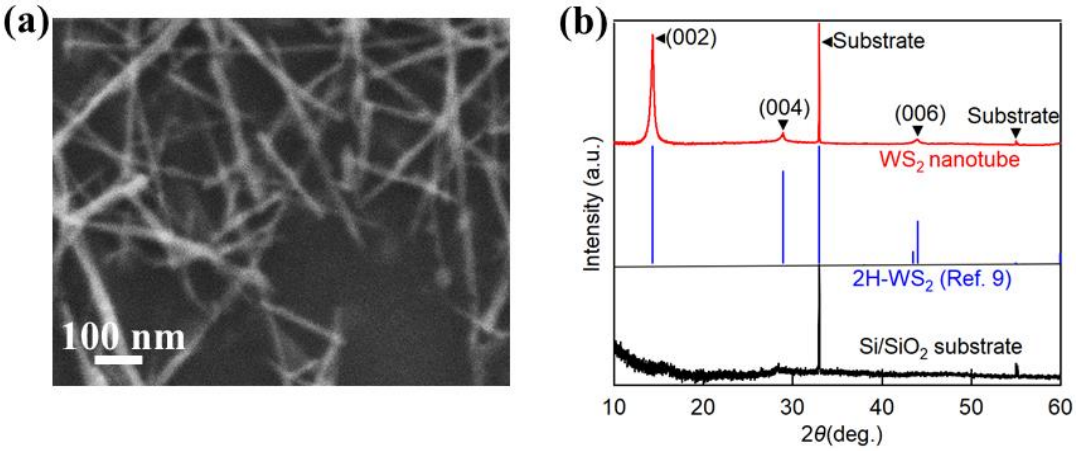

Fig. S7. (a) The FESEM image of our synthesized WS_2 nanotubes on the Si/SiO_2(300nm) substrate, which was taken by JSM-7100F (JEOL Ltd.). As mentioned in the main text, to avoid side-reactions, the tungsten oxide nanowires on a Si substate, which were synthesized by our CVD processes, were dispersed into ethanol solution, and then the nanowire dispersed solution was dropped on a Si/SiO_2 substate, and then the tungsten oxide nanowires on the substate were converted to the WS_2 nanotubes. Therefore, the morphology of the WS_2 nanotube networks is different from that of the tungsten oxide nanowires (Fig.S2(a)). The FESEM image indicates the presence of nano-flakes is negligible. (b) The XRD patterns of WS_2 nanotubes, 2H-WS_2, and the Si/SiO_2 substrate. The XRD patterns of the WS_2 nanotubes and Si/SiO_2 substrate were taken by the Rigaku SmartLab diffractometer with $CuK_{\alpha 1}$ (1.5406 Å) radiation. The XRD pattern of the 2H-WS_2 is depicted using data in a literature[9]. The XRD patten of the WS_2 nanotubes correspond to the 2H-WS_2 [9]. However, the *d* value of (002) is estimated to be 6.192 Å, which is slightly larger than that of bulk 2H-WS_2, 6.180 Å, and such lattice expansion in the WS_2 nanotubes has been discussed in previous studies[10,11].

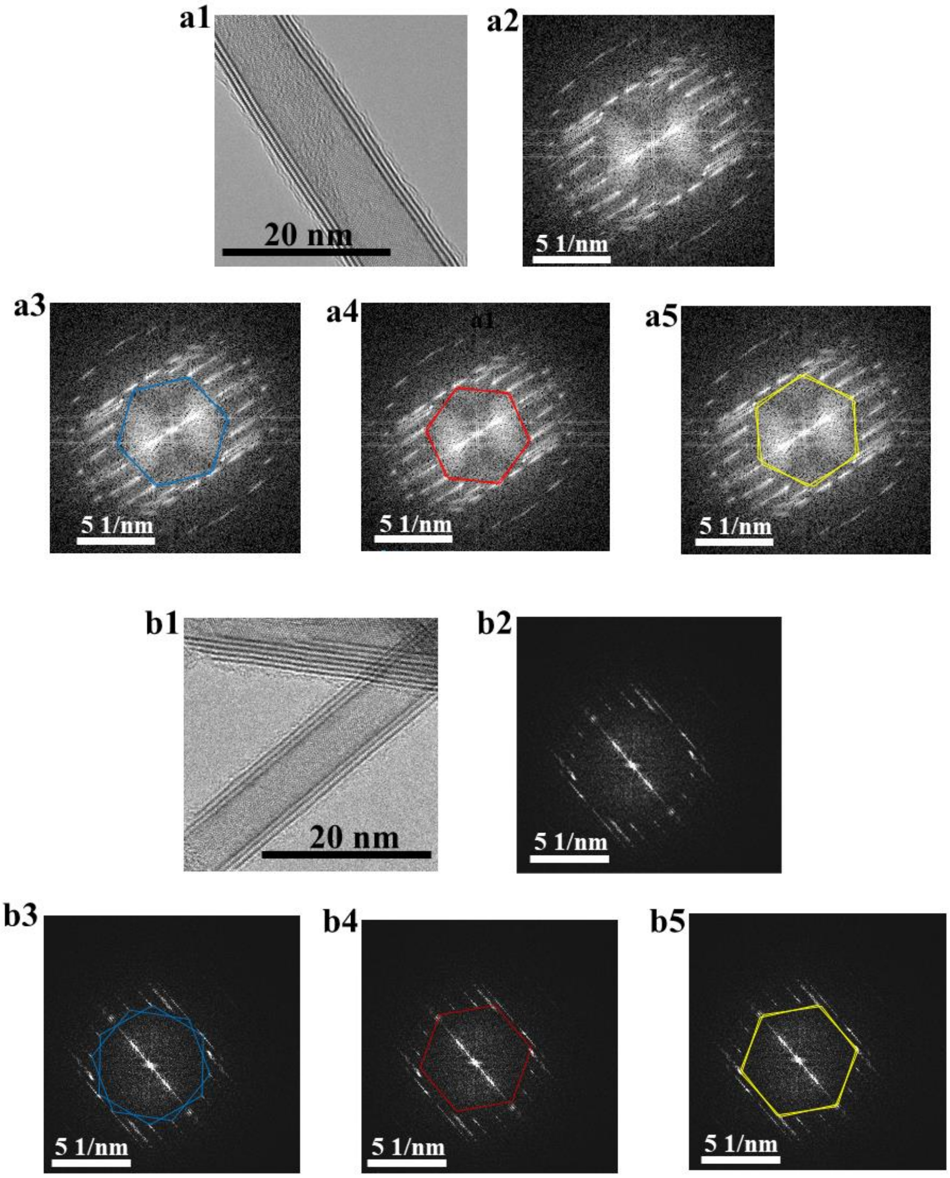

Fig. S8. Evaluation of chiralities of the WS_2 nanotubes. (a1) The TEM image of the WS_2 nanotubes with three walls obtained in this study, and (a2) its FFT pattern. From the FFT patterns, we identified three different chiralities with chiral angles of 10° (blue hexagon, a3), 9° (red hexagon, a4), and 27° (yellow, a5), respectively. The TEM image of another WS_2 nanotubes with three walls (b1), and its FFT pattern (b2). From the FFT pattern, we identified the following three nanotubes with different chiral angles of 10° (blue hexagon, b3), armchair (red hexagon, b4), and 27° (yellow hexagon, b5), respectively. These results indicate that the different walls exhibit different chiralities.

Preparation of WS_2 Flakes:

We prepared the dispersion of WS_2 flakes using liquid phase exfoliation according to the method used in a previous study [12,13]. WS_2 flake dispersions were prepared by sonicating powder (WS_2, Sigma-Aldrich) at a concentration of 20 g L^{-1} using tip-type ultra-sonic (Sonifiner, Branson Co.) for 0.5 hours at 25% amplitude in 20 mL of deionized water. After that, the dispersed solution was centrifuged at 3600g for 1-hour and then the supernatant was discarded. The sediment was redispersed in a 20 mL surfactant solution containing 1% sodium cholate (SC, Sigma-Aldrich). This dispersion was then sonicated at 25% amplitude for 1 hour. This dispersion was sonicated, and then centrifuged at 1000g for 30 minutes with a swinging bucket rotor. The sediment at the 10% level was collected after centrifugation, and the supernatant was discarded. To perform an optical measurement, a sample of the sediment was washed with methanol and re-dispersed.

UV-vis extinction and absorption measurement:

We checked the optical absorption spectrum of L-WS_2 NTs by using UV-vis spectrophotometer with an integrating sphere (UV-3600i Plus, Shimazu Co.). For this measurement, the L-WS_2 NTs sample dispersed in methanol solution were dropped on quartz substrate and thin films of L-WS_2 NTs was prepared. Fig. S9 indicates the extinction spectrum (blue curve), which was obtained in L-WS_2 NTs dispersed solution in methanol using a UV-Vis spectrometer without an integrating sphere, and the absorption spectrum of the thin film of the same sample (red curve). In the absorption spectrum, we can clearly identify the A-exciton peak of the sample to be 1.97 eV.

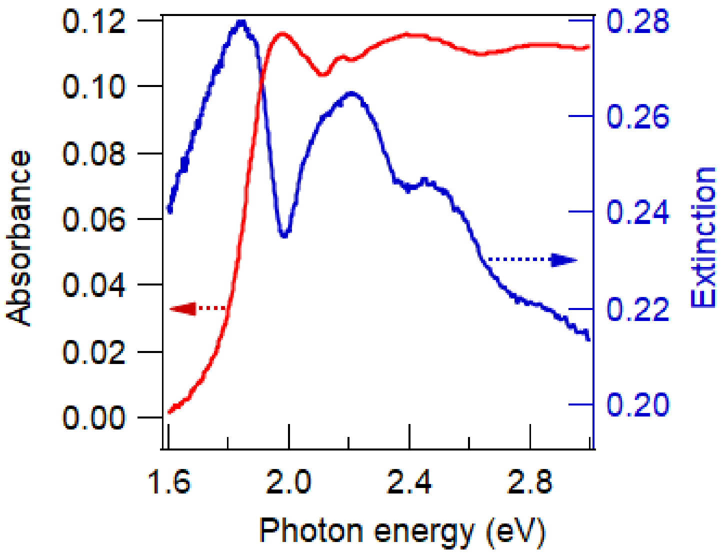

Fig. S9. The absorption (red) spectra of L-WS_2 NTs in thin film and the extinction (blue) spectra of L-WS_2 NTs in methanol solution.

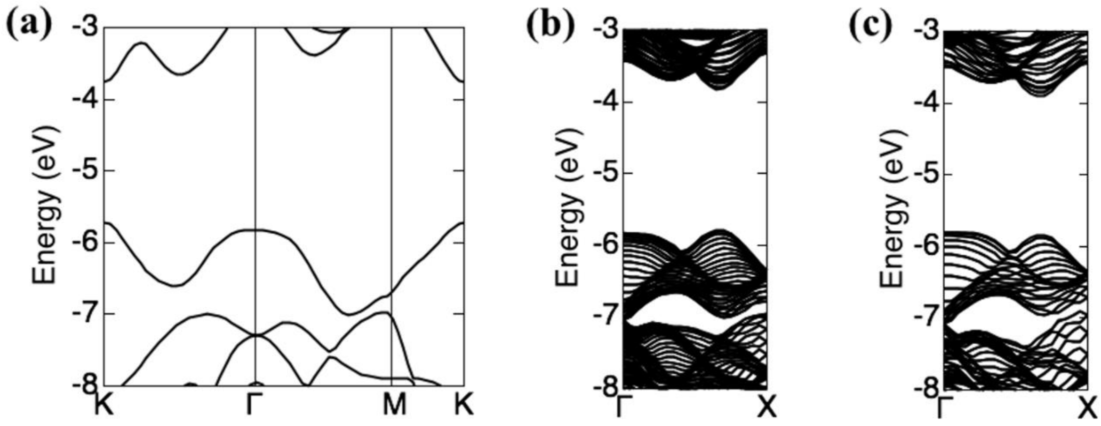

Fig. S10. Electronic energy bands of (a) monolayer, (b) (28,28) nanotube, and (c) (17,17) nanotube of WS_2. Energies are measured from the vacuum level.

Table S1. Diameter comparison with previously [14,15] prepared WS_2 nanotubes.

Nanotubes	Average Diameter (nm)
WS_2 (Ref. 14)	Outermost diameter: 20.3 ± 5.2<br>Innermost diameter: 7.7 ± 1.1
WS_2 (Ref. 15)	Outermost diameter: 20.0 ± 2.3<br>Innermost diameter: 7.1 ± 1.6
WS_2 (This work)	Outermost diameter: 13 ± 4<br>Innermost diameter: 6 ± 3

References:

1. Lamire, M., Labbe, P., Goreaud, M. & Raveau, B. Refining and new analysis of $W_{18}O_{49}$ structure. *Rev. Chim. Miner.* **24,** 369-381 (1987).

2. Hong, K., Xie, M., Hu, R. & Wu, H. Synthesizing tungsten oxide nanowires by a thermal evaporation method. *Appl. Phys. Lett.* **90,** 173121 (2007).

3. Hong, K., Xie, M., Hu, R. & Wu, H. Diameter control of tungsten oxide nanowires as grown by thermal evaporation. *Nanotechnology*. **19,** 085604 (2008).

4. Lu, C.H., Hon, M.H., Kuan, C.Y. & Leu, C. A complementary electrochromic device based on $W_{18}O_{49}$ nanowire arrays and Prussian blue thin films. *RSC Adv.* **6,** 1913-1918 (2016).

5. Liu, B.J.W. *et al.* Ultrathin $W_{18}O_{49}$ nanowire assemblies for electrochromic devices. *Nano lett.* **13,** 3589-3593 (2013).

6. Wu, J. *et al.* Change in crystalline structure of $W_{18}O_{49}$ nanowires induced by X-ray irradiation and its effects on field emission. *RSC Adv.* **8,** 752-760 (2018).

7. Li, W., Sun, J., Zhang, J., Ganiyat, O.A. and Cui, Y. Facile fabrication of $W_{18}O_{49}$/PEDOT: PSS/ITO-PET flexible electrochromic films by atomizing spray deposition. *Surf. Interfaces*. **2,** 100002 (2021).

8. Nethravathi, C. *et al.* Chemical unzipping of WS_2 nanotubes. *Acs Nano.* **7,** 7311-7317 (2013).

9. H.E. Swanson, *et al.* Circular of the Bureau of Standards No. 539 Volume 8: Standard X-ray Diffraction Powder Patterns, US Department of Commerce, National Institute of Standards and Technology, (Page no. 65-66) 1959.

10. Zak, A. *et al.* Scaling up of the WS_2 nanotubes synthesis. *Fuller. Nanotub. Carbon Nanostructures*. **19,** 18-26 (2010).

11. Sinha, S.S. *et al.* MoS_2 and WS_2 Nanotubes: Synthesis, Structural Elucidation, and Optical Characterization. *J. Phys. Chem. C.* **125,** 6324-6340 (2021).

12. Backes, C. *et al.* Production of highly monolayer enriched dispersions of liquid-exfoliated nanosheets by liquid cascade centrifugation. *ACS Nano*. **10,** 1589-1601 (2016).

13. Griffin, A. *et al.* Effect of surfactant choice and concentration on the dimensions and yield of liquid-phase-exfoliated nanosheets. *Chem. Mater.* **32,** 2852-2862 (2020).

14. Yomogida, Y., Miyata, Y. & Yanagi, K. Transistor properties of relatively small-diameter tungsten disulfide nanotubes obtained by sulfurization of solution-synthesized tungsten oxide nanowires. *Appl. Phys. Express.* **12,** 085001 (2019).

15. Rahman, M. A. *et al.* Improved synthesis of WS_2 nanotubes with relatively small diameters by tuning sulfurization timing and reaction temperature. *Jpn. J. Appl. Phys.* **60,** 100902 (2021).
